# Anticoagulation therapy in patients with chronic obstructive pulmonary disease in the acute exacerbation stage

**DOI:** 10.3892/etm.2013.1001

**Published:** 2013-03-12

**Authors:** XIAOSHAN SHI, HONGYUN LI

**Affiliations:** Department of Respiratory Medicine, The Fifth Affiliated Hospital of Zhengzhou University, Zhengzhou, Henan 450052, P.R. China

**Keywords:** chronic obstructive pulmonary disease, anticoagulant therapy, pulmonary function, blood coagulation function, fibrinogen, D-dimer

## Abstract

This aim of this study was to investigate the effects of intervention with low-molecular-weight heparin calcium on the pulmonary function and blood coagulation function of patients with chronic obstructive pulmonary disease (COPD) in the acute exacerbation stage. A total of 70 patients with COPD in the acute exacerbation stage were selected and randomly divided into the control group (32 cases) and the anticoagulation group (38 cases). The patients of the control group were treated with oxygen therapy, spasmolysis, asthmarelief, cough relief and sputum reduction. The patients in the anticoagulation group, in addition to the conventional therapy used the control group, were also treated with low-molecular-weight heparin calcium 4100 AXaIU/time. by abdominal subcutaneous injection once every 12 h. For both groups, one treatment course lasted 10 days. The pulmonary function indicators and blood coagulation indicators of the two groups before and after treatment were determined. Following treatment, the pulmonary function and arterial blood gas indicators of the two groups were significantly better than those before treatment, and the extents of the improvements in the anticoagulation group were significantly greater than those in the control group. The D-dimer level and blood coagulation indicators [international normalized ratio (INR), prothrombin time (PT), activated partial thromboplastin time (APTT) and fibrinogen (FIB) concentration] of the anticoagulation group were significantly improved compared with those prior to treatment, but there were no significant differences between these measures before and after treatment for patients in the control group. No serious adverse reaction occurred in the anticoagulation group. Although, the conventional therapy was able to improve the overall condition of patients with COPD, it was not able to effectively improve the hypercoagulative state of the patients’ blood. The combination of conventional therapy with low-molecular-weight heparin calcium anticoagulation improved the blood coagulation and pulmonary functions of patients.

## Introduction

Chronic obstructive pulmonary disease (COPD) is a common disease in the respiratory system and has extremely high morbidity and mortality rates. Due to the progressive decline of pulmonary function, COPD seriously affects the quality of life of patients and their ability to work. At present, clinical treatments for COPD in the acute exacerbation stage mainly focus on oxygen therapy, spasmolysis, asthma and cough relief, sputum reduction and the application of large amounts of antibiotics. This treatment usually ignores the potential existence of hypercoagulation and pulmonary arterial hypertension in patients. Studies have shown that there is a prethrombotic state in patients with COPD in which the blood is in a hypercoagulable and hyperfibrinolytic state (1). Persistent progression may cause pulmonary microthromboembolisms to form (2), which are important causes of the formation and aggravation of pulmonary arterial hypertension. For patients with acute exacerbations of chronic obstructive pulmonary disease (AECOPD), there is a significant correlation between blood coagulation and anticoagulation, and between oxidation and antioxidation (3,4). Early application of anticoagulant may lead to an improved prognosis (5). In recent years, The Fifth Affiliated Hospital of Zhengzhou University (Zhengzhou, China) has used low-molecular-weight heparin calcium to conduct anticoagulation intervention for patients with COPD in the acute exacerbation stage. As a result, it was identified that such treatments are able to significantly improve the pulmonary function of patients, slow disease progress, reduce the hospitalization period and hospitalization frequency, and obtain a better result.

## Subjects and methods

### Subjects

A total of 70 patients with COPD in the acute exacerbation stage who were hospitalized in the respiratory medicine department from January 2009 to December 2010 and complied with the inclusion criteria, were selected and randomly divided into two groups: the anticoagulation group and the control group. In the anticoagulation group, there were 38 cases, including 23 males and 15 females. Their mean age was 58.6±5.4 years old, mean body mass index was 26.3±5.1 kg/m^2^ and mean disease course was 14.1±3.8 years. With regard to complications, there were 7 cases of hypertension, 6 cases of coronary heart disease and 4 cases of diabetes mellitus. In the control group, there were 32 cases, including 19 males and 13 females. Their mean age was 57.9±5.2 years old, mean body mass index was 25.9±4.9 kg/m^2^ and mean disease course was 13.8±4.0 years. With regard to complications, there were 5 cases of hypertension, 5 cases of coronary heart disease and 3 cases of diabetes mellitus. Between the two groups, there were no significant differences in gender, age, body mass index, disease course, complications or blood gas (all P>0.05). This study was conducted in accordance with the Declaration of Helsinki and with approval from the Ethics Committee of the Fifth Affiliated Hospital of Zhengzhou University. Written informed consent was obtained from all participants.

### Inclusion criteria

i) The disease history, physical examination, pulmonary function and chest X-ray examination of patients all complied with the diagnostic criteria of the Diagnosis and Treatment Guidelines of Chronic Obstructive Pulmonary disease revised by the Respiratory Disease Branch of the Chinese Medical Association in 2007 (6). There was a pulmonary function abnormality, obstructive ventilatory disorder: namely after bronchodilator was inhaled, the forced expiratory volume in 1 sec (FEV1) was <50% of the predicted value. In addition, there were clinical signs of respiratory failure or right heart failure, and the ratio of FEV1 to forced vital capacity (FVC; FEV1/FVC) was <70%. Furthermore, the disease condition was in the acute exacerbation stage. ii) Patients were not treated with anticoagulants such as aspirin, warfarin and heparin in the month before inclusion; iii) Under the agreement and coordination of patients and their families, signed informed consent was provided.

### Exclusion criteria

i) Bronchial asthma, rhinitis, pulmonary tuberculosis, lung cancer, pulmonary embolism as well as complicated pneumothorax or pleural effusion. ii) Thrombocytopenia, hemorrhagic tendency and disturbances of blood coagulation. iii) Acute bacterial endocarditis, severe liver and renal insufficiency, cerebrovascular sequelae and other systemic severe complications. iv) Patients with an allergic history to heparin drugs. v) Patients who were not able to adhere to treatment or administer drugs in accordance with the provisions due to any factor.

### Treatment methods

The patients in the control group were treated conventionally with continuous low-flow oxygen uptake, anti-infection treatment, bronchodilation, phlegm elimination, heart failure correction, etc. In addition to the conventional therapy of the control group, low-molecular-weight heparin calcium (Subilin; Hangzhou Sanofi-Synthelabo Minsheng Pharmaceutical Company, Hangzhou, China; SDA Approval Word J20090005) 4100 AXaIU/time. was also administered to patients in the treatment group by abdominal subcutaneous injection once every 12 h. One course of treatment lasted 10 days.

### Detection items and methods

For the two groups of patients, the following investigations were conducted once before treatment and again after 10 days of treatment: i) pulmonary function detection: FVC, FEV1 and the ratio of FEV1 to FVC (FEV1/FVC%) were determined with a MasterScreen Impulse Oscillometry Spirometer (MSIOS) model professional pulmonary function testing instrument manufactured by Erich Jaeger GmbH (Höchberg, Germany); ii) blood gas analysis: oxygen saturation of blood (SaO_2_), partial pressure of oxygen (PaO_2_) and partial pressure of carbon dioxide (PaCO_2_) were determined using the blood gas analyzer [Model: BXY4-GEM 3000; IL (instruments) Company, Boston, MA, USA], and oxygen uptake was stopped for half an hour prior to blood sampling. iii) D-dimer (D-D) determination: an enzyme-linked immunosorbent assay (ELISA) double antibody sandwich method was used. The reagents were provided by Shanghai Jiemen Biotechnology Corp (Shanghai, China). Assays were strictly conducted in accordance with the manufacturer’s instructions. The sensitivity of this method was 10 *μ*g/l, and the normal range was <300 *μ*g/l. iv) Determinations of prothrombin time (PT), activated partial thromboplastin time (APTT) and fibrinogen (FIB) concentration: an ACL200 Automated Coagulation Analyzer (Beckman-Coulter Inc., Miami, FL, USA) and the kit manufactured by Stago Inc. (Parsippany, NJ, USA) was used. v) Adverse reactions following administration were monitored.

### Statistical analysis

Data analysis was conducted using SPSS 11.0 statistical software, and measurement data were expressed as mean ± standard deviation (± sd) and analyzed by Student’s t-test. Enumeration data were expressed as a percentage and analyzed by χ^2^ test. P<0.05 was considered to indicate a statistically significant result.

## Results

### Pulmonary function and arterial blood gas indicators

Prior to treatment, there were no significant differences in pulmonary function indicators (FEV1, FEV1/FVC) and arterial blood gas indicators (SaO_2_, PaO_2_, PaCO_2_) between the two groups (t=0.5036, 0.9799, 1.3240, 1.6131, 1.1870, all P>0.05), and there was a significant difference between the two groups. Following treatment, the pulmonary function indicators and arterial blood gas indicators of the two groups were significantly improved compared with those before treatment (in the anticoagulation group: t=8.2064, 6.9207, 9.4351, 7.5845, 7.5241; in the control group t=5.5588, 3.0795, 3.9374, 2.8488, 2.7726, all P<0.01). However, the extents of the improvements of the anticoagulation group were significantly greater than those of the control group (t=3.2732, 4.0167, 7.3392, 2.2528, 3.0008, all P<0.01; Table I).

### Blood coagulation indicators

Prior to treatment, no significant differences in the D-D level and blood coagulation indicators [international normalized ratio (INR), PT, APTT, FIB) between the groups were observed (t=0.1066, 1.8234, 0.6094, 0.8251, 0.4514; all P>0.05), and there was a significant difference between the two groups. Following the treatment, the D-D level and blood coagulation indicators of the anticoagulation group were significantly improved compared with those prior to treatment (t=5.7207, 23.235, 25.318, 23.841, 11.354; all P<0.01), and the extents of the improvements were significantly greater than those in the control group (t=7.7376, 19.365, 23.373, 21.008, 9.7121; all P<0.01). For the control group, there was no significant difference between the D-D level and blood coagulation indicators prior to and following treatment (t=1.9409, 8.0000, 1.1592, 1.6562, 1.2726; all P>0.05; Table II).

### Adverse reactions

In the anticoagulation group, there was no thrombocytopenia and no anaphylaxis. One case presented skin ecchymosis at the abdominal injection site, but no special treatment was conducted. After 7 days, the skin ecchymosis naturally disappeared and no other bleeding situation was observed. In addition, 9 cases presented mild prolongation of APTT, and the range was between 46 and 60 sec (normal reference value: 30–45 sec).

## Discussion

For patients with COPD, chronic hypoxia and hypercapnia stimulate hematopoietic function enhancement of the bone marrow to induce a compensatory increase in polycythemia and elevations in blood viscosity and loop resistance (7) as well as abnormal blood rheology. In addition, dehydration, a reduction in erythrocyte deformability, slow blood flow and acid-base imbalance cause the blood to be in a hypercoagulative state. The above pathological changes cause erythrocytes to gather into a mass and form a thrombus which promotes adhesion and the aggregation of platelets and endothelium, damages the vascular endothelium, initiates the blood coagulation process and causes blood coagulation state abnormalities in the blood of patients with COPD (8). Prolongations of PT, APTT and INR are predominant in the coagulation state abnormalities and D-D and FIB levels are significantly elevated (9,10). There may be multiple pulmonary small artery thrombi *in situ*, which readily cause pulmonary arterial hypertension. Hematocrit and D-D are positively correlated with pulmonary arterial systolic pressure, indicating that the hypercoagulative state is the key factor causing pulmonary heart disease (11). There are a number of indicators reflecting the hypercoagulative state of blood. Among them, i) APTT is an important indicator of blood coagulation function, and APTT prolongation indicates a reduction in a blood coagulation factor (prothrombin, FIB, VIII, IX, XI) or an increase in an anticoagulation substance. ii) FIB is a protein of high molecular weight with a blood coagulation function that is synthesized by the liver and is a precursor of fibrin. As a blood coagulation factor, FIB increases blood viscosity and increases platelet aggregation by influencing platelet-specific receptors. Elevation of the FIB level is an important risk factor of thrombosis. It is an indicator of prethrombotic state, airway progressive inflammation and lung tissue injury (12). iii) D-D is a specific degradation product formed by the hydrolysis of cross-linked fibrin by plasmin and it is the smallest peptide generated by cross-linked fibrin degradation. Due to a good stability, high sensitivity and strong specificity in plasma, D-D has been recognized as a mark of thrombosis or thromboclasis (13), and it is helpful for clinical anticoagulation therapy, thrombolytic therapy and the judgment of efficacy and prognosis. Previous studies (14) have confirmed that the D-D level is correlated with illness severity and that a change in the D-D level may act as an indicator for evaluating AECOPD severity and treatment efficacy, and that the D-D level is correlated with the prognosis and pulmonary function improvement of AECOPD (15).

Heparin is a strong anticoagulant and acts through anti-reactivator II and anti-reactivator X to relieve the hyper-coagulative state and prevent pulmonary artery thrombosis. Heparin reduces blood cell cohesion and blood viscosity, reduces pulmonary circulation resistance, improves pulmonary circulation and systemic micro-circulation and aids gas exchange and histanoxia improvement. In addition, heparin may prevent blood platelets from releasing 5-HT, bradykinin and other media and is able to relieve bronchospasm and thus reduces airway resistance, improves ventilatory function, and corrects hypoxia and CO_2_ retention to control respiratory failure. As an anticoagulant, heparin has a reliable efficacy in the clinic, but its long-term application easily causes a bleeding risk due to its high anticoagulative activity. Therefore, its applications are limited. Relevant data show that low-molecular-weight heparin calcium is an indirect thrombin inhibitor obtained by heparin lysis or purification, and it maintains potent anti-Xa and anti-IIa activities (16). In addition, its antithrombotic activity is higher, but its anticoagulative activity is weaker. As low-molecular-weight heparin calcium plays an antithrombotic role, it causes less thrombocytopenia and bleeding. In addition, its subcutaneous bioavailability is high and it is easily absorbed when administered by subcutaneous injection. Its half-life is ∼24 h, it is easily administered by a single daily subcutaneous injection, patient pain is low, it is inexpensive and the strict monitoring of blood coagulation indicators is unnecessary (17–20).

The present study shows that i) after low-molecular-weight heparin calcium anticoagulation therapy, the extents of the improvements in the pulmonary function and arterial blood gas indicators of the anticoagulation group are significantly greater than those of the control group, and the extents of the improvements in D-D level and blood coagulation indicators of the anticoagulation group are also significantly greater than those of the control group. ii) Blood coagulation activity of patients with COPD is significantly enhanced and is in the hypercoagulative state (D-D and FIB elevations are predominant), confirming that the hypercoagulative state in the body is a result of the common action of FIB and activated endogenous blood coagulation route. iii) SaO_2_ and PaO_2_ are negatively correlated with D-D and FIB, and PaCO_2_ is correlated with D-D and FIB, suggesting that hypoxemia and hypercapnia are closely associated with changes in D-D and FIB levels. iv) Following low-molecular-weight heparin calcium anticoagulation therapy, patients do not present evident adverse reactions, confirming its safety. In conclusion, the supplementation of conventional therapy with low-molecular-weight heparin calcium is able to improve more effectively the blood coagulation state of patients with COPD in the acute exacerbation stage, improve blood rheology indicators, reduce blood viscosity and improve pulmonary circulation and thus improve cardiopulmonary function and reduce complications (21). In addition, adverse reactions are few and mild. Therefore, low-molecular-weight heparin calcium shall be regarded as the preferred anticoagulation drug for treating COPD in the acute exacerbation stage.

## Figures and Tables

**Table I:** Comparisons of pulmonary function indicators and arterial blood gas indicators of the two groups before and after treatment (mean ± sd).

Groups	Time of measurement	FEV1 (liter)	FEV1/FVC (%)	SaO_2_ (%)	PaO_2_ (mmHg)	PaCO_2_ (mmHg)
Anticoagulation (n=38)	Before treatment	1.36±0.17	62.3±8.7	90.8±4.9	71.5±4.3	52.2±6.4
	After treatment	1.89±0.36^ab^	78.4±11.4^ab^	98.6±1.4^ab^	78.4±3.6^ab^	41.9±5.5^ab^
Control (n=32)	Before treatment	1.34±0.16	60.2±9.2	92.3±4.5	73.2±4.5	50.2±7.7
	After treatment	1.64±0.26^a^	67.8±10.5^a^	95.7±1.9^a^	76.3±4.2^a^	45.7±5.0^a^

a P<0.01, compared with before treatment;

b P<0.01, compared with the control group. FEV1, forced expiratory volume in 1 sec; FVC, forced vital capacity; SaO_2_, oxygen saturation of blood; PaO_2_, partial pressure of oxygen; PaCO_2_, partial pressure of carbon dioxide.

**Table II:** Comparisons of blood coagulation state of the two groups of patients before and after treatment (mean ± sd).

Groups	Time of measurement	D-D level (*μ*g/l)	INR	PT (sec)	APTT (sec)	FIB (g/l)
Anticoagulation (n=38)	Before treatment	482.8±253.6	0.87±0.05	9.6±0.67	28.8±1.08	3.97±0.84
	After treatment	237.4±74.9^ab^	1.36±0.12^ab^	13.7±0.74^ab^	35.7±1.42^ab^	2.16±0.51^ab^
Control (n=32)	Before treatment	476.5±237.2	0.85±0.04	9.5±0.70	29.0±0.92	3.88±0.82
	After treatment	389.5±89.6	0.93±0.04	9.7±0.68	29.4±1.01	3.63±0.75

a P<0.01, compared with before treatment;

b P<0.01, compared with the control group. D-D, D-dimer; INR, international normalized ratio; PT, prothrombin time; APTT, activated partial thromboplastin time; FIB, fibrinogen.

## References

[1] Hu XY, Li Y, Du YC, Xu JY (2009). Prethrombolic state and efficacy of anticoagulation of patients with acute exacerbations of chronic obstructive pulmonary disease. International Journal of Respiration.

[2] Jiang ZH, Lv YW, Sang HY, Zhu MY, Zhu ZP, Ding P (2007). The study of prethrombotic state in patients with chronic respiratory failure study. Chinese Journal of Emergency Medicine.

[3] Huang J, Liu XJ, Bao HR (2011). Chronic obstructive pulmonary disease in patients with coagulation dysfunction and oxidative stress in the relationship between. J Int Med.

[4] Huang J, Liu XJ, Bao HR, Zhang Y, Tan EL, Liao JM (2011). The relationship between coagulation/anticoagulation imbalance and oxidative stress in patients with chronic obstructive pulmonary disease. Zhonghua Nei Ke Za Zhi.

[5] Wang PF, Ling CH (2010). Correlation study of patients with chronic obstructive pulmonary disease (COPD) arterial blood gas analysis and plasma D-dimer. Hemorheol.

[6] Chronic Obstructive Pulmonary Disease Committee, Respiratory Society, Chinese Medical Association (2007). Improved confidence, right awareness and rational treatment: opening up a new era of prevention and treatment of chronic obstructive pulmonary disease. Zhonghua Jie He He Hu Xi Za Zhi.

[7] Zarfis JH (2005). Atherosclerosis thrombosis and inflammatory risk factors from history and the laboratory to real life. Eur Heart J.

[8] Ye XF, Liu S, Yang JH (2008). Pulmonary heart disease in patients with D-dimer levels and systolic pulmonary artery pressure and carbon dioxide partial pressure correlation analysis. Cardiopulmonary Vascular J.

[9] Yu CN, Sun L, Jiang P (2008). Patients with pulmonary heart disease coagulation and fibrinolysis state. Zhonghua Jian Yan Yi Xue Za Zhi.

[10] Duan JM, An X, Zhang JY, Xu F, Liu P (2008). Chronic obstructive pulmonary disease (COPD) patients with hypoxemia clinical D-dimer testing and clinical analysis. Journal of Clinical Pulmonary Medicine.

[11] Wu WN, Wang LX, Chen SX (2009). Patients with chronic obstructive pulmonary disease (COPD) erythrocyte sedimentation rate, hematocrit, D-dimer, pulmonary artery systolic pressure. Practical J Med.

[12] Dahl M, Tybjaerg-Hansen A, Vestbo J, Lange P, Nordestgaard BG (2001). Elevated plasma fibrinogen associated with reduced pulmonary function and increased risk of chronic obstructive pulmonary disease. Am J Respir Crit Care Med.

[13] Wu TP (2007). Significance in patients with chronic obstructive pulmonary disease D-dimer. Chin Med.

[14] Yu QH, Li YC (2010). Chronic obstructive pulmonary disease in patients with D-dimer and antithrombin relationship with prognosis. Tianjin Traditional Chin Med.

[15] Mo FP

[16] Wu ZX, Zhang CF, Wang W, Li ZQ, Wang BF, Xu H (2011). Therapeutic Effect of LMWH for Acute Exacerbation Patients of Pulmonary Heart Disease. China Health Industry.

[17] Zhang Y, Li YX, Cao Y, Chai YL, Niu XQ (2012). Unfractionated heparin and low-molecular-weight heparin in AECOPD pulmonary embolism in clinical observation. Journal of Kunming Medical University.

[18] Segal JB, Streiff MB, Hofmann LV, Thornton K, Bass EB (2007). Management of venous thromboembolism: a systematic review for a practice guideline. Ann Intern Med.

[19] Chen LY, Ying KJ, Hong WJ, Zhou P (2005). Comparison of low-molecular-weight-heparin and unfractionated heparin for acute PTE. J Zhejiang Univ Sci B.

[20] Wang R, Peng HX (2008). Low-molecular-weight heparin and heparin sodium efficacy for the treatment of pulmonary heart disease with comparison - report of 40 cases. New Med.

[21] Qiu XY, Jin N (2008). Anticoagulant treatment of severe chronic obstructive pulmonary disease oxygenation function and central venous pressure impact. Chin Modern Sci Magazine.

